# Maximum Correntropy with Variable Center Unscented Kalman Filter for Robust Power System State Estimation

**DOI:** 10.3390/e24040516

**Published:** 2022-04-06

**Authors:** Zhenglong Sun, Chuanlin Liu, Siyuan Peng

**Affiliations:** 1 Key Laboratory of Modern Power System Simulation and Control & Renewable Energy Technology, Northeast Electric Power University, Ministry of Education, Jilin 132012, China; nedusunzl@neepu.edu.cn (Z.S.); 2019301011120@neepu.edu.cn (C.L.); 2 School of Information Engineering, Guangdong University of Technology, Guangzhou 510006, China

**Keywords:** correntropy with variable center, unscented Kalman filter, robustness, power system state estimation

## Abstract

The robust Kalman filter with correntropy loss has received much attention in recent years for forecasting-aided state estimation in power systems, since it efficiently reduces the negative influence of various abnormal situations, such as non-Gaussian communication, changing environment, and instrument failures, and obviously improves the stability of power systems. However, the existing correntropy-based robust Kalman filters usually use the Gaussian function with a fixed center as the kernel function in correntropy, which may not be a suitable choice in practical applications of power system forecasting-aided state estimation (PSSE). To address this issue, a new and robust unscented Kalman filter, called the maximum correntropy with variable center unscented Kalman filter (MCVUKF), is proposed in this paper for PSSE. Specifically, MCVUKF adopts an extended version of correntropy, whose center can be located at any position, to replace the original correntropy in an unscented Kalman filter to improve the performance in PSSE. Moreover, by using an exponential function of the innovation vector to adjust a covariance matrix, an enhanced MCVUKF (En-MCVUKF) method is also developed for suppressing the influence of bad data to the innovation vector and further improving the accuracy of PSSE. Finally, extensive simulations have been conducted on IEEE 14-bus, 30-bus, and 57-bus test power systems, and the simulation results have shown the superiority of the proposed MCVUKF and En-MCVUKF methods compared with several related state-of-the-art Kalman filter methods.

## 1. Introduction

State estimation has played an important role in the energy management system, which is necessary for the reliability, safety, economic operation, and resiliency of power systems. Generally speaking, the state estimation in power systems can be classified into two categories, including static state estimation (SSE) and forecasting-aided state estimation (FASE) [1]. Different from SSE that captures the real-time operating state of the power system, FASE mainly focuses on predicting the trend of the power system. Due to the variety of loads in the power system, which usually leads to the change of the state information of buses, SSE fails to deal with the influence of the change of system loads. By contrast, based on the obtained prior information, FASE can analyze and predict the changing trend of the power system. Hence, in recent years, FASE has gained considerable attention in the field of power systems for state estimation [2,3].

As one of the most useful power system forecasting-aided state estimation (PSSE) techniques, the Kalman-type filters and their improvements have been widely applied in FASE due to their excellent tracking ability in power systems [4,5,6]. Traditional Kalman filters (KF) aim to obtain the accurate state estimation for a linear dynamic system, which are difficult to achieve the optimal filter for nonlinear systems. However, in many practical applications, the power systems are usually nonlinear. To address the nonlinear filtering issue, two typical nonlinear extensions of the traditional KF, i.e., extended Kalman filter (EKF) [7,8] and unscented Kalman filter (UKF) [9,10], have been widely proposed. For example, Zhao et al. proposed an $H_{\infty }$ EKF method for PSSE, which was based on the control theory to bound the influence of the uncertainties, such as uncertain inputs or varying generator transient reactance in different operation conditions [11]. Generally speaking, to estimate the state of power system, the EKF methods aim at using the first-order Taylor expansion to approximate the nonlinear functions, which seriously suffer from the strong nonlinearities of the model. In order to overcome this drawback, the UKF methods have been successfully developed in PSSE due to the good performance in highly nonlinear systems and a simple calculation process. In fact, the UKF approaches mainly utilize the unscented transformation (UT) to approximate the probability distribution functions. It is worth noting that most of the existing KF methods are proposed by using the popular minimum means square error (MMSE) criterion, because it can achieve the optimal performance under Gaussian assumption. However, the distribution of noise in various practical applications usually fails to satisfy the condition of Gaussian assumption [12,13,14].

Due to unknown system inputs (e.g., tripping of customer loads and parameter variations), the power system model is subject to uncertainties. Moreover, the process and observation noise in power systems are usually non-Gaussian with multi-peak or heavy-tailed distribution, which is verified by the Pacific Northwest National Lab [15]. In this situation, the performance of the MMSE criterion-based KF methods may degrade seriously in PSSE. In order to improve the robustness of traditional KF methods, several information theoretic criteria have been adopted as the robust criterion to replace the original MMSE criterion in KF algorithms [16,17,18,19,20]. Typical examples include the minimum error entropy criterion (MEE) [21,22,23] and the maximum correntropy criterion (MCC) [24,25,26]. For instance, Ma et al. respectively proposed a adaptive extended Kalman filter with correntropy loss [27] and a unscented Kalman filter with the generalized correntropy loss (GCL-UKF) [28] for the real-time state estimation of the power system; Chen et al. first utilize the MEE criterion into UKF and proposed a minimum error entropy-based UKF method for robust power system state estimation [29]. Although the MEE and MCC-based KF methods have been demonstrated to be effective for suppressing the bad impact of the heavy-tailed noise and outliers in the power system, they still have some drawbacks. For example, the MEE-based KF methods usually require high computational complexity in PSSE. The MCC-based KF methods have a reasonable computational cost in practical tasks. However, the kernel function of correntropy used in MCC-based KF methods is restricted to the zero-mean Gaussian function, which may not be a superior selection in practical power system applications. The main reason is that when the error distribution in a power system is non-zero mean, the performance of the error criteria located at zero may decline obviously.

Toward this end, in this paper, a novel and robust unscented Kalman filter—namely, the maximum correntropy with variable center unscented Kalman filter (MCVUKF)—is proposed, which is used to enhance the performance in PSSE. As an extended version of the original correntropy, the correntropy with variable center, has a flexible center that can be located at any position, which can match well the error distribution for various practical situations [30,31]. Instead of using the traditional MMSE and MCC criteria, the proposed MCVUKF method utilizes the maximum correntropy with variable center criterion to the UKF to improve the estimation accuracy and enhance the stability against non-Gaussian noise. Moreover, to further improve the performance of PSSE, an enhanced MCVUKF (En-MCVUKF) method is also developed by using an exponential function of the innovation vector to adjust a covariance matrix to suppress the effect of abnormal data to the innovation vector. Finally, extensive simulations have been conducted on IEEE 14-bus, 30-bus, and 57-bus test power systems, and the simulation results have shown the good performance of the proposed MCVUKF and En-MCVUKF methods compared with several related state-of-the-art traditional and robust KF methods.

The rest of this paper is organized as follows. In Section 2, we briefly describe the model of power system and the correntropy with variable center. The proposed MCVUKF and En-MCVUKF methods are presented in Section 3. Extensive simulations are conducted in Section 4. Finally, the conclusion is given in Section 5.

## 2. Related Work

In this section, the model of the power system is first introduced. Then, the definition of the correntropy with a variable center is presented.

### 2.1. Model of the Power System

A general nonlinear dynamical power system can be described by a set of continuous-time nonlinear differential and algebraic equations, which are frequently expressed in the following discrete-time state space form at time instance *i*:

(1) $$x_{i}=f\left(x_{i-1}\right)+w_{i}$$

(2) $$y_{i}=h\left(x_{i}\right)+v_{i}$$

where $x_{i}\in R^{n\times 1}$ responds to the state vector that consists of the subvectors of nodal voltage magnitude $V_{i}\in R^{1\times n_{1}}$ and nodal voltage angles $\theta_{i}\in R^{1\times n_{2}}$ with $n=n_{1}+n_{2}$; f(·) is the state-transition function that relates $x_{i}$ to $x_{i-1}$; $w_{i}\in R^{n\times 1}$ stands for the system process noise with covariance matrix $Q_{i}\in R^{n\times n}$; $y_{i}\in R^{n\times 1}$ denotes the measurement vector; h(·) is the measurement function that consists of the subvectors of the real power injection $T_{a}\in R^{1\times m_{1}}$, the reactive power injection $Z_{a}\in R^{1\times m_{2}}$, the real power flow $T_{ab}\in R^{1\times m_{3}}$, and the reactive power flow $Z_{ab}\in R^{1\times m_{4}}$ with $m=m_{1}+m_{2}+m_{3}+m_{4}$; $v_{i}\in R^{n\times 1}$ stands for the measurement noise with covariance matrix $R_{i}\in R^{n\times n}$. Generally speaking, in a practical power system model, the distributions of the existing noises are usually non-Gaussian. In this work, the main purpose is to develop the new and robust KF method. Based on Holt’s two-parameter linear exponential smoothing technique [1], the function f(·) can be expressed in the following form:

(3) $$x_{i}=a_{i-1}+b_{i-1}$$

(4) $$a_{i-1}=\alpha_{i-1}x_{i-1}+\left(1-\alpha_{i-1}\right)x_{i-1}^{*}$$

(5) $$b_{i-1}=\beta_{i-1}\left(a_{i-1}-a_{i-2}\right)+\left(1-\beta_{i-1}\right)b_{i-2}$$

where the parameters $\alpha_{i-1}$ and $\beta_{i-1}$ are in [0,1], $x_{i-1}^{*}$ stands for the predicted state vector at time instance i−1. Here, the state forecasting function (3) is utilized in (1) to predict the state vector in advance when the state prediction of the KF method is executed. Based on the standard real power and reactive power balance as well as power flow equations, the function h(·) is defined as follows [28,29]:

(6) $$T_{a}=|V_{a}|\sum_{b=1}^{N}\left|V_{b}\right|\left(G_{ab}\cos\theta_{ab}+B_{ab}\sin\theta_{ab}\right)$$

(7) $$Z_{a}=|V_{a}|\sum_{b=1}^{N}\left|V_{b}\right|\left(G_{ab}\sin\theta_{ab}-B_{ab}\cos\theta_{ab}\right)$$

(8) $$T_{ab}=V_{a}^{2}\left(G_{ga}+G_{ab}\right)-|V_{a}\left|\right|V_{b}|\left(G_{ab}\cos\theta_{ab}+B_{ab}\sin\theta_{ab}\right)$$

(9) $$Z_{ab}=-V_{a}^{2}\left(B_{ga}+B_{ab}\right)-|V_{a}\left|\right|V_{b}|\left(G_{ab}\sin\theta_{ab}-B_{ab}\cos\theta_{ab}\right)$$

where $T_{a}$ and $Z_{a}$, respectively, stand for the real and reactive power injection at bus *a*, $V_{a}$ stands for the voltage magnitude at bus *a*, $\theta_{ab}$ denotes the voltage angle between buses *a* and *b*, $T_{ab}$ and $Z_{ab}$, respectively, denote the real power flow and reactive power flow between buses *a* and *b*. $G_{ab}$ and $B_{ab}$ denote the conductance and susceptance of the line between buses *a* and *b*, and $G_{ga}$ and $B_{ga}$ denote the conductance and susceptance of the shunt at bus *a*.

### 2.2. Correntropy with Variable Center

As one of the commonly used robust similarity measures, correntropy is first proposed in information theoretical learning (ITL) [32]. Compared with the traditional squared Euclidean distance (SED), correntropy can effectively reduce the bad impact of non-Gaussian noise and outliers. Due to its simplicity and robustness, in recent decades, correntropy has been successfully used in many practical tasks [33,34,35]. Considering two random variables *A* and *B*, the definition of correntropy is given by [32,36]:

(10) $$V_{\sigma }\left(A,B\right)=E\left[G_{\sigma }\left(A-B\right)\right]=E\left[G_{\sigma }\left(e\right)\right]=\int G_{\sigma }\left(a-b\right)dF_{AB}\left(a,b\right)$$

where $G_{\sigma }\left(\cdot \right)$ is a kernel function, σ is the kernel bandwidth parameter, and $F_{AB}\left(a,b\right)$ represents the joint distribution function of (A,B) and e=A−B. In most cases, the Gaussian kernel used in correntropy-based robust methods is usually the Gaussian kernel function with the center located at zero because it has some outstanding advantages such as strict positive-definiteness, simplicity, and smoothness. However, in many real-world environments, the zero-mean Gaussian function is often not a good selection for correntropy, since it fails to match well the error distribution in practical applications.

To overcome this drawback, correntropy with a variable center has been proposed in recent years [30], whose definition is expressed as follows:

(11) $$V_{\sigma ,c}\left(A,B\right)=E\left[G_{\sigma }\left(e-c\right)\right]=E\left[\frac{1}{\sqrt{2\pi }\sigma }\exp\left(-\frac{{\left(e-c\right)}^{2}}{2\sigma^{2}}\right)\right]$$

where *c* denotes the center location. It is worth noting that when c=0, the correntropy with a variable center will reduce to the original correntropy. Since the join distribution $F_{AB}\left(a,b\right)$ is usually unknown in real-world applications, based on a finite number of data points ${\left\{\left(a_{n},b_{n}\right)\right\}}_{n=1}^{N}$, the estimation of the correntropy with a variable center is:

(12) $$\begin{array}{c} {\hat{V}}_{N,\sigma ,c}\left(A,B\right)=\frac{1}{N}\sum_{n=1}^{N}G\left(a_{n}-b_{n}-c\right) \end{array}$$

where ${\hat{V}}_{N,\sigma ,c}$ stands for the estimator of $V_{\sigma ,c}$, and *N* is the number of samples. Using the Taylor series expansion of the Gaussian kernel, we can rewrite (11) as follows:

(13) $$\begin{array}{c} V_{\sigma ,c}\left(A,B\right)=\frac{1}{\sqrt{2\pi }\sigma }\sum_{n=0}^{\infty }\frac{{\left(-1\right)}^{n}}{2^{n}n!}E\left[\frac{{\left(e-c\right)}^{2n}}{2\sigma^{2n}}\right] \end{array}$$

Similar to the original correntropy, one can observe that from the viewpoint of statistics, the correntopy with a variable center still can capture the higher order moments (i.e., all the even order moments) of *e* that are useful to reduce the sensitivity to non-Gaussian noise and outliers. Therefore, the correntopy with a variable center is robust to non-Gaussian noise and outliers.

## 3. Maximum Correntropy with Variable Center Unscented Kalman Filter for Robust State Estimation

In this section, the maximum correntropy criterion with variable center (MCC-VC) is used in the UKF, and the maximum correntropy with variable center unscented Kalman filter (MCVUKF) approach is derived based on the discrete-time state space Equations (1) and (2) in a nonlinear power system. The derivation process of MCVUKF includes time update and measurement update.

### 3.1. MCVUKF

#### 3.1.1. Time Update

Based on the unscented transformation technique, combining the estimated state ${\hat{x}}_{i-1|i-1}$ and the covariance matrix $P_{i-1|i-1}$, a sequence of 2n+1 sigma points can be generated:

(14) $$X_{i-1|i-1}^{s}=\left\{\begin{array}{ccc} {\hat{x}}_{i-1|i-1}, & \; & s=0\\ {\hat{x}}_{i-1|i-1}+{\left(\sqrt{\left(n+\lambda \right)P_{i-1|i-1}}\right)}_{s}, & \; & s=1,\ldots ,n\\ {\hat{x}}_{i-1|i-1}+{\left(\sqrt{\left(n+\lambda \right)P_{i-1|i-1}}\right)}_{s-n}, & \; & s=n+1,\ldots ,2n \end{array}\right.$$

where ${\left(\sqrt{\left(n+\lambda \right)P_{i-1|i-1}}\right)}_{s}$ denotes the *s*-th column vector of the square root matrix, λ is the scalar parameter with $\lambda =\delta^{2}\left(n+\kappa \right)-n$ in which δ∈[0,1] determines the diffusion degree of the sigma point around, and the parameter κ=3−n is used to reduce the higher order errors of the mean and the covariance approximations. Assuming that $x_{0}$ is an initial state variable with the initial state mean ${\hat{x}}_{0|0}=E\left[x_{0}\right]$, we have the initial state estimate error covariance matrix $P_{0|0}$ in the following form:

(15) $$P_{0|0}=E\left[\left(x_{0}-{\hat{x}}_{0}\right){\left(x_{0}-{\hat{x}}_{0}\right)}^{T}\right]$$

where ${\left[\cdot \right]}^{T}$ denotes the transpose operator. Afterward, we obtain ${\hat{x}}_{i|i-1}$ and $P_{i|i-1}$:

(16) $${\hat{x}}_{i|i-1}=\sum_{s=0}^{2n}\omega_{\eta }^{i}f\left(X_{i-1|i-1}^{s}\right)$$

(17) $$P_{i|i-1}=\sum_{s=0}^{2n}\omega_{\nu }^{i}\left(f\left(X_{i-1|i-1}^{s}\right)-{\hat{x}}_{i|i-1}\right){\left(f\left(X_{i-1|i-1}^{s}\right)-{\hat{x}}_{i|i-1}\right)}^{T}+Q_{i-1}$$

where $\omega_{\eta }^{i}$ and $\omega_{\nu }^{i}$, respectively, denote the weights of the sigma point mean with $\omega_{\eta }^{0}=\frac{\lambda }{n+\lambda }$, $\omega_{\nu }^{0}=\frac{\lambda }{n+\lambda }$, and $\omega_{\eta }^{i}=\omega_{\nu }^{i}=\frac{1}{2(n+\lambda )},\;\;i=1,2,\ldots ,2n$, in which λ is related to the distribution of the state variable.

#### 3.1.2. Measurement Update

Similar to the time update process, 2n+1 sigma points should be calculated in the measurement update process from ${\hat{x}}_{i|i-1}$ and $P_{i|i-1}$:

(18) $$X_{i-1|i-1}^{s}=\left\{\begin{array}{ccc} {\hat{x}}_{i-1|i-1}, & \; & s=0\\ {\hat{x}}_{i-1|i-1}+{\left(\sqrt{\left(n+\lambda \right)P_{i|i-1}}\right)}_{s}, & \; & s=1,\ldots ,n\\ {\hat{x}}_{i-1|i-1}+{\left(\sqrt{\left(n+\lambda \right)P_{i|i-1}}\right)}_{s-n}, & \; & s=n+1,\ldots ,2n \end{array}\right.$$

After that, we have the prior mean ${\hat{y}}_{i|i-1}$ and the predicted measurement cross-covariance matrix $P_{xy,i}$:

(19) $${\hat{y}}_{i|i-1}=\sum_{s=0}^{2n}\omega_{\eta }^{i}h\left(X_{i|i-1}^{s}\right)$$

(20) $$P_{xy,i}=\sum_{s=0}^{2n}\omega_{\nu }^{i}\left(X_{i|i-1}^{s}-{\hat{x}}_{i|i-1}\right){\left(h\left(X_{i|i-1}^{s}\right)-{\hat{y}}_{i|i-1}\right)}^{T}$$

In this work, we utilize the maximum correntropy criterion with a variable center to a statistical linear regression model for completing the measurement update. Firstly, we define a prior state estimation error $\eta (x_{i})$ and a measurement slope matrix $H_{i}$ as follows:

(21) $$\eta \left(x_{i}\right)=x_{i}-{\hat{x}}_{i|i-1}$$

(22) $$H_{i}={\left(P_{i|i-1}^{-1}P_{xy,i}\right)}^{T}$$

Based on (21) and (22), we can approximate (2) in the following form:

(23) $$y_{i}\approx {\hat{y}}_{i|i-1}+H_{i}\left(x_{i}-{\hat{x}}_{i|i-1}\right)+v_{i}$$

Combining (1), (21) and (23), we obtain:

(24) $$\left[\begin{array}{c} {\hat{x}}_{i|i-1}\\ {\hat{y}}_{i}-{\hat{y}}_{i|i-1}+H_{i}{\hat{x}}_{i|i-1} \end{array}\right]=\left[\begin{array}{c} I\\ H_{i} \end{array}\right]x_{i}+\xi_{i}$$

where I denotes a unity matrix, $\xi_{i}={\left[-\eta \left(x_{i}\right),v_{i}\right]}^{T}$ and

(25) $$E\left[\xi_{i}\xi_{i}^{T}\right]=\left[\begin{array}{ccc} S_{p,i|i-1}S_{p,i|i-1}^{T} & \; & 0\\ 0 & \; & S_{r,i}S_{r,i}^{T} \end{array}\right]=S_{i}S_{i}^{T}$$

in which $S_{i}$ is the Cholesky decomposition factor of the matix $E\left[\xi_{i}\xi_{i}^{T}\right]$. Obviously, we derive by multiplying $S_{i}^{-1}$ in both sides of (24):

(26) $$S_{i}^{-1}\left[\begin{array}{c} {\hat{x}}_{i|i-1}\\ {\hat{y}}_{i}-{\hat{y}}_{i|i-1}+H_{i}{\hat{x}}_{i|i-1} \end{array}\right]=S_{i}^{-1}\left[\begin{array}{c} I\\ H_{i} \end{array}\right]x_{i}+S_{i}^{-1}\xi_{i}\Rightarrow D_{i}=W_{i}x_{i}+E_{i}$$

where

(27) $$D_{i}=S_{i}^{-1}\left[\begin{array}{c} {\hat{x}}_{i|i-1}\\ {\hat{y}}_{i}-{\hat{y}}_{i|i-1}+H_{i}{\hat{x}}_{i|i-1} \end{array}\right]$$

(28) $$W_{i}=S_{i}^{-1}\left[\begin{array}{c} I\\ H_{i} \end{array}\right]$$

(29) $$E_{i}=S_{i}^{-1}\xi_{i}$$

with $D_{i}=\left[d_{1,i},\ldots ,d_{L,i}\right]$, $W_{i}=\left[w_{1,i},\ldots ,w_{L,i}\right]$, and $E_{i}=\left[e_{1,i},\ldots ,e_{L,i}\right]$. Clearly, based on $E\left[\xi_{i}\xi_{i}^{T}\right]=S_{i}S_{i}^{T}$ and (29), we have $E\left[E_{i}E_{i}^{T}\right]=I$. Due to

(30) $$e_{k,i}=d_{k,i}-w_{k,i}x_{i}$$

where $e_{k,i}$ denotes the *i*-th element of $e_{i}$, the maximum correntropy criterion with a variable center is used to obtain the optimal values of the state variables, and the optimization problem is given by

(31) $$\arg\max_{x_{i}}\frac{1}{\sqrt{2\pi }\sigma }\left[\frac{1}{L}\sum_{k=1}^{L}\exp\left(-\frac{{\left(e_{k,i}-c\right)}^{2}}{2\sigma^{2}}\right)\right]$$

where *L* denotes the number of data samples. Based on (31), we have the optimal estimate of $x_{i}$ by minimizing the following optimization problem:

(32) $$\begin{array}{c} {\hat{x}}_{i}=\arg\min_{x_{i}}J\left(x_{i}\right)=\frac{1}{\sqrt{2\pi }\sigma }\left[\exp\left(-\frac{c^{2}}{2\sigma^{2}}\right)-\frac{1}{L}\sum_{k=1}^{L}\exp\left(-\frac{{\left(e_{k,i}-c\right)}^{2}}{2\sigma^{2}}\right)\right] \end{array}$$

Then, the partial derivative of $J(x_{i})$ with respect to $x_{i}$ is derived as follows:

(33) $$\begin{array}{c} \frac{\partial J(x_{i})}{x_{i}}=-\frac{1}{\sqrt{2\pi }\sigma }\times \frac{1}{2\sigma^{2}}\frac{1}{L}\sum_{k=1}^{L}\exp\left(-\frac{{\left(e_{k,i}-c\right)}^{2}}{2\sigma^{2}}\right)\left(d_{k,i}-w_{k,i}x_{i}\right)w_{k,i} \end{array}$$

By setting $\frac{\partial J(x_{i})}{x_{i}}=0$, we have

(34) $$\begin{array}{c} x_{i}={\left(\sum_{k=1}^{L}\exp\left(-\frac{{\left(e_{k,i}-c\right)}^{2}}{2\sigma^{2}}\right)w_{k,i}^{T}w_{k,i}\right)}^{-1}\left(\sum_{k=1}^{L}\exp\left(-\frac{{\left(e_{k,i}-c\right)}^{2}}{2\sigma^{2}}\right)w_{k,i}^{T}d_{k,i}\right) \end{array}$$

Obviously, (34) is a fixed-point equation with respect to $x_{i}$. According to [28], it can be further rewritten in the form of matrix:

(35) $$\begin{array}{c} x_{i}={\left(W_{i}^{T}C_{i}W_{i}\right)}^{-1}\left(W_{i}^{T}C_{i}D_{i}\right) \end{array}$$

where

(36) $$C_{i}=\left[\begin{array}{ccc} C_{x,i} & \; & 0\\ 0 & \; & C_{y,i} \end{array}\right]$$

(37) $$C_{x,i}=diag\left\{\exp\left(-\frac{{\left(e_{1,i}-c\right)}^{2}}{2\sigma^{2}}\right),\ldots ,\exp\left(-\frac{{\left(e_{n,i}-c\right)}^{2}}{2\sigma^{2}}\right)\right\}$$

(38) $$C_{y,i}=diag\left\{\exp\left(-\frac{{\left(e_{n+1,i}-c\right)}^{2}}{2\sigma^{2}}\right),\ldots ,\exp\left(-\frac{{\left(e_{n+m,i}-c\right)}^{2}}{2\sigma^{2}}\right)\right\}$$

Combining (25) and (28) yields

(39) $$W_{i}=\left[\begin{array}{ccc} S_{p,i|i-1}^{-1} & \; & 0\\ 0 & \; & S_{r,i}^{-1} \end{array}\right]\left[\begin{array}{c} I\\ H_{i} \end{array}\right]=\left[\begin{array}{c} S_{p,i|i-1}^{-1}\\ S_{r,i}^{-1}H_{i} \end{array}\right]$$

Assume that $S_{p}$, $S_{r}$, $C_{x}$, and $C_{y}$ stand for $S_{p,i|i-1}$, $S_{r,i}$, $C_{x,i}$, and $C_{y,i}$ respectively. One can easily derive by using (36) and (39):

(40) $${\left(W_{i}^{T}C_{i}W_{i}\right)}^{-1}={\left({\left({\left(S_{p}\right)}^{-1}\right)}^{T}C_{x}S_{p}^{-1}+H_{i}^{T}{\left({\left(S_{p}\right)}^{-1}\right)}^{T}C_{y}S_{p}^{-1}H_{i}\right)}^{-1}$$

Based on the matrix inversion lemma, (40) can be rewritten as:

(41) $$\begin{array}{cc} {\left(W_{i}^{T}C_{i}W_{i}\right)}^{-1}= & {\left(A+BDC\right)}^{-1}=S_{p}C_{x}^{-1}S_{p}^{T}-S_{p}C_{x}^{-1}S_{p}^{T}H_{i}\times \\ \; & {\left(H_{i}{\left(S_{p}\right)}^{-1}S_{p}C_{x}^{-1}S_{p}^{T}H_{i}^{T}+S_{r}C_{y}^{-1}S_{r}^{T}\right)}^{-1}H_{i}S_{p}C_{x}^{-1}S_{p}^{T} \end{array}$$

where ${\left({\left(S_{p}\right)}^{-1}\right)}^{T}C_{x}S_{p}^{-1}=A$, $H_{i}^{T}=B$, ${\left({\left(S_{p}\right)}^{-1}\right)}^{T}C_{y}S_{p}^{-1}=C$, and $H_{i}=D$. Using the definition of *a*, one can obtain:

(42) $$\begin{array}{c} D_{i}=\left[\begin{array}{c} S_{p,i|i-1}^{-1}{\hat{x}}_{i|i-1}\\ S_{r}^{-1}\left(y_{i}-h\left({\hat{x}}_{i|i-1}\right)+H_{i}{\hat{x}}_{i|i-1}\right) \end{array}\right] \end{array}$$

Moreover, combining (36) and (42), we have

(43) $$\begin{array}{cc} W_{i}^{T}C_{i}D_{i}= & {\left(S_{p}^{-1}\right)}^{T}C_{x}S_{p}^{-1}{\hat{x}}_{i|i-1}+H_{i}^{T}{\left(S_{p}^{-1}\right)}^{T}C_{y}S_{r}^{-1}\left(y_{i}-h\left({\hat{x}}_{i|i-1}\right)+H_{i}{\hat{x}}_{i|i-1}\right) \end{array}$$

Finally, one can derive the following update rule by using (35) and (43):

(44) $$\begin{array}{c} x_{i}={\hat{x}}_{i|i-1}+{\hat{K}}_{i}\left(y_{i}-{\hat{y}}_{i|i-1}\right) \end{array}$$

where

(45) $$\begin{array}{c} {\hat{K}}_{i}={\hat{P}}_{i|i-1}H_{i}^{T}\left(H_{i}{\hat{P}}_{i|i-1}H_{i}^{T}+R_{i}^{-1}\right) \end{array}$$

(46) $$\begin{array}{c} {\hat{P}}_{i|i-1}={\hat{S}}_{p,i|i-1}C_{x,i}^{-1}{\left({\hat{S}}_{p,i|i-1}\right)}^{T} \end{array}$$

(47) $$\begin{array}{c} R_{i}={\hat{S}}_{r,i}C_{y,i}^{-1}{\left({\hat{S}}_{r,i}\right)}^{T} \end{array}$$

Furthermore, we also derive the corresponding update rule for the covariance matrix:

(48) $$\begin{array}{c} {\hat{P}}_{i|i}=\left(I-{\hat{K}}_{i}H_{i}\right){\hat{P}}_{i|i-1}{\left(I-{\hat{K}}_{i}H_{i}\right)}^{T}+{\hat{K}}_{i}R_{i}{\hat{K}}_{i}^{T} \end{array}$$

#### 3.1.3. Optimization of the Free Parameters σ and *c*

For the proposed MCVUKF method, there are two important parameters (e.g, the kernel width σ and the center location *c*) that have great influence on the performance of MCVUKF in PSSE. Particularly, the parameter σ controls all robust properties of the correntropy with a variable center, and the parameter *c* enhances the learning performance of the correntropy with a variable center in various practical applications with non-zero-mean error distribution. Similar to [30,31], an efficient method is adopted in this subsection to optimize the two parameters by using the following optimization problem:

(49) $$\begin{array}{c} \left\{\sigma (i),c(i)\right\}=\arg\min_{\sigma \in T,c\in C}\frac{1}{\sqrt{2\pi }\sigma }\left[\exp\left(-\frac{c^{2}}{2\sigma^{2}}\right)-\frac{1}{L}\sum_{k=1}^{L}\exp\left(-\frac{{\left(e_{k,i}-c\right)}^{2}}{2\sigma^{2}}\right)\right] \end{array}$$

where σ(i) and c(i) denote the adapted parameters at iteration time *i*, and T and C, respectively, stand for the admissible sets of parameters σ and *c*. In fact, many approaches can be adopted to solve the above optimization problem. In this work, for the kernel width parameter σ, a traditional gradient-based approach is utilized to optimize the optimization problem (49) for selecting the appropriate value in each iteration. Moreover, for the parameter *c*, in order to simplify the computation complexity of the proposed method, we set the center location *c* in MCVUKF to the mean or median value of the error by using the Parzen window theory. The update rule of *c* can be expressed as follows:

(50) $$\begin{array}{c} c\left(i\right)=median\left\{|e_{1,i}\left|,\right|e_{2,i},|,\ldots ,e_{L,i}\left|\right|\right\} \end{array}$$

It is remarkable that when the window length is large enough, the obtained value of the parameter *c* is suitable for the error curve, whose efficiency can be observed in the simulation results.

### 3.2. En-MCVUKF

Due to anomaly conditions, the measurements usually include large error, which may affect the estimation performance of the proposed MCVUKF method in the power system [28,37]. In order to avoid the performance degradation and improve the reliability of the state estimation for MCVUKF, an enhanced MCVUKF (En-MCVUKF) method is developed, which can be considered as a potential weighting method to solve the state estimation problems compared with traditional approaches. Specifically, the En-MCVUKF method uses an exponential function of the innovation vector to adjust a covariance matrix such that the estimation performance can be maintained. The main reason is that when the absolute residual vector increases because of unwanted disturbances, this exponential weight function will efficiently restrict the magnitude of the residual and suppress the negative influence of bad data to the estimation performance in PSSE. Motivated by the idea in [28,37], we also apply an exponential function to the proposed MCVUKF method for updating the covariance matrix, which is given by:

(51) $$\begin{array}{c} R_{i}=R_{i}\exp\left(-|y_{i}-h\left(x_{i}\right)|\right) \end{array}$$

(52) $$\begin{array}{c} R_{i}=R_{i}^{-1} \end{array}$$

Obviously, when (51) and (52) are utilized to replace the (47) in the proposed MCVUKF approach for updating $R_{i}$, the En-MCVUKF approach is derived. Next, based on the above-derived procedure, the detailed steps of the En-MCVUKF algorithm for state estimation in a power system can be summarized in Algorithm 1:
**Algorithm 1** En-MCVUKF Algorithm for Power System State Estimation**Input:** An initial estimation of state vector ${\hat{x}}_{0|0}=E\left[x_{0}\right]$ with the covariance matrix $P_{0}$, two initial values for free parameters σ and *c*, a small enough positive value ε, and the number of max iterations maxIter, set i=0.
**Output:** The estimation of state vector ${\hat{x}}_{i|i,t}$ and the posterior covariance matrix $P_{i|i}$.
1:**repeat**2:      Calculate the prior estimation of ${\hat{x}}_{i|i-1}$ and $P_{i|i-1}$ by using (16) and (17), and $S_{p,i|i-1}$ by using the Cholesky decomposition.3:      Calculate the prior measurement ${\hat{y}}_{i|i-1}$ by using (18)–(20), and the measurement slope matrix $H_{i}$ by using (22).4:      Build the statistical linear regression model by using (24).5:      Transform (23) into (26), and set t=1 and the initial value of ${\hat{x}}_{i|i,t}$ as ${\hat{x}}_{i|i,0}$ at time instant t=06:      **repeat**7:            Calculate the residual error $e_{k,i}$ by using:
(53)$$e_{k,i}=d_{k,i}-w_{k,i}{\hat{x}}_{i|i,t-1}$$8:            Calculate the covariance matrix $R_{i}$ by using (51) and (52):9:            Update ${\hat{x}}_{i|i}$ by using the following equation:
(54)$$\begin{array}{c} {\hat{x}}_{i|i,t}={\hat{x}}_{i|i-1}+{\hat{K}}_{i}\left(y_{i}-{\hat{y}}_{i|i-1}\right) \end{array}$$10:            t=t+1.11:      **until** $\frac{∥{\hat{x}}_{i|i,t}-{\hat{x}}_{i|i,t-1}∥}{∥{\hat{x}}_{i|i,t-1}∥}>\varepsilon$12:      Calculate $P_{i|i}$ by using (48).13:      i=i+1.14:**until** i>maxIter15:Return The estimation of state vector ${\hat{x}}_{i|i,t}$ and the posterior covariance matrix $P_{i|i}$.

## 4. Numerical Results

### 4.1. Simulation Settings

**Figure 1:**
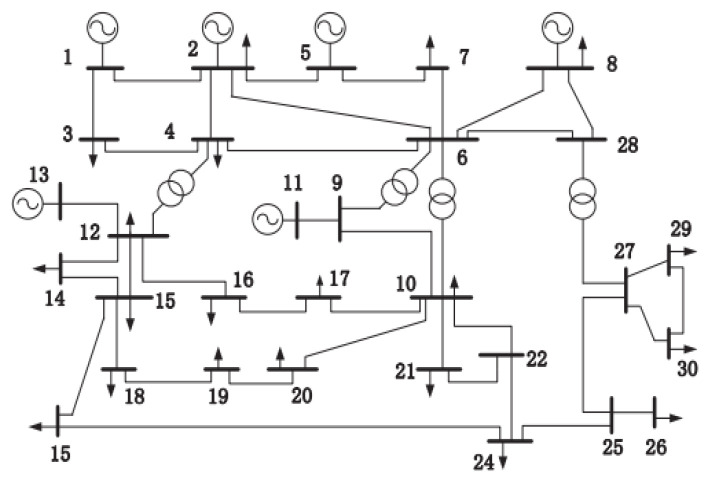
Structure of the IEEE 30 bus test system.

In the simulations, three test systems, including the IEEE 14-bus system, the IEEE 30-bus system, and the IEEE 57-bus system are adopted for PSSE to illustrate the effectiveness and superiority of the proposed MCVUKF and En-MCVUKF algorithms under different types of non-Gaussian noises. Figure 1 shows the structure of the IEEE 30-bus test system [29]. The data used for the simulation are the power system data, which are obtained from the University of Washington Power System Test Case Archive (http://www.ee.washington.edu/research/pstca/, accessed on 13 March 2022). In order to demonstrate the filtering performance of the proposed algorithms, three different scenarios are used in the simulations [28].

Moreover, two commonly used performance metrics, i.e., the performance index *J* (in p.u.) and the absolute error of the phase angle and amplitude of each node voltage [37], are utilized to evaluate the filtering performance of the proposed methods in the simulations. Particularly, the smaller the values of *J* (in p.u.) and absolute error, the better the filtering performance. Due to the fact that the used evaluation metrics penalize different properties in the power system for PSSE, extensive simulation results are reported on these diverse criteria to achieve a comprehensive filtering performance evaluation for different methods.

To show the effectiveness and robustness of MCVUKF and En-MCVUKF in PSSE, we also make a comparison between the proposed methods and the five most related methods, which are EKF [8], UKF [38], EnUKF [28], MCCEKF [17], and MCUF [16]. Specifically, EKF, UKF, and EnUKF are the traditional KF methods that are derived by using the MMSE criterion, and MCCEKF and MCUF are the robust KF methods by using the MCC criterion. Furthermore, all methods are conducted on the platform of MATLAB 2018a running on i9-10900K and 3.70-GHz CPU. Without otherwise mentioned, we set the initial state error covariance matrix to be 0.0001I. We implement 100 random trials for all comparison methods to achieve the reliable simulations results, and all simulations results are obtained by calculating the average results on every test system.

### 4.2. Case 1: Non-Gaussian Noise with Outliers in Measurements

In this subsection, we conduct simulation on the practical power system with the measurement noises corrupted by non-Gaussian noise with random outliers. Here, two widely used non-Gaussian noise models (i.e., mixed-Gaussian noise model and Laplace noise model) are adopted in this work, whose detailed descriptions are given by [22]:

1. Mixed-Gaussian noise model: The model for the mixed-Gaussian noise is expressed as:
  (55) $$\begin{array}{c} \left(1-\theta \right)N\left(\lambda_{1},\upsilon_{1}^{2}\right)+\theta N\left(\lambda_{2},\upsilon_{2}^{2}\right) \end{array}$$
  where $N\left(\lambda_{i},\upsilon_{i}^{2}\right)\left(i=1,2\right)$ denotes the Gaussian distributions with mean values $\lambda_{i}$ and variances $\upsilon_{i}^{2}$, and θ stands for the mixture coefficient. Usually, $\upsilon_{2}^{2}\gg \upsilon_{1}^{2}$. Hence, the parameter vector for the mixed Gaussian noise is defined as $V_{mix}=\left(\lambda_{1},\lambda_{2},\upsilon_{1}^{2},\upsilon_{2}^{2},\theta \right)$.
2. Laplace noise model: The Laplace noise is distributed with probability density function (PDF):
  (56) $$\begin{array}{c} p\left(\upsilon \right)=\frac{1}{2}\exp^{-|\upsilon |} \end{array}$$

**Figure 2:**
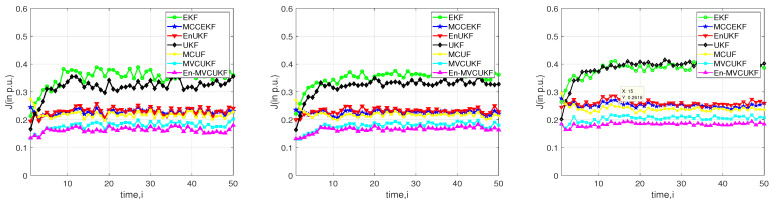
Filtering performance index *J* (in p.u.) of the proposed methods and EKF, MCCEKF, EnUKF, UKF, and MCUF under mixed-Gaussian measurement noise with $V_{mix}=\left(0,0,1,100,0.15\right)$ in three test systems. (**Left**) IEEE 14-bus test system; (**Middle**) IEEE 30-bus test system; (**Right**) IEEE 57-bus test system.

First, we use the mixed-Gaussian noise with the noise parameter vector $V_{mix}=\left(0,0,1,100,0.15\right)$ to model the measurement noises in three test systems. The simulation results in terms of the performance index *J* (in p.u.) are shown in Figure 2 for the test systems at each time instant *i*. From this figure, one can draw the following conclusions:

- The overall filtering performance of the proposed MVCUKF and En-MVCUKF methods is the best and the second best in three used test power systems when compared with five state-of-the-art methods, indicating that the proposed method is of superiority. The main reasons can be summarized as follows:
  (1) Compared with the MMSE criterion-based KF methods (i.e., EKF, UKF, and EnUKF), the proposed methods adopt the robust maximum correntropy criterion with a variable center to suppress the bad influence of the heavy-tailed non-Gaussian noise in PSSE;
  (2) Compared with the MCC-based robust KF methods (i.e., MCCEKF and MCUF) that have a fixed center in correntropy, the MVCUKF and En-MVCUKF methods utilize an enhanced version of the traditional correntropy whose center can be located at any position; thus, they can be more robust to the non-zero-mean non-Gaussian noise;
- In the same situation, the original MCC and MCC with variable center-based KF methods can achieve better filtering performance than the MMSE-based KF methods, which indicates the robustness of correntropy and its enhanced version used in the KF model.
- Clearly, the enhanced KF methods (e.g., EnUKF and En-MVCUKF) usually have slightly better filtering performance than the original UKF and MVCUKF methods. That demonstrates that using an exponential function of the innovation vector to adjust a covariance matrix can effectively improve the filtering performance in power systems for state estimation.

**Figure 3:**
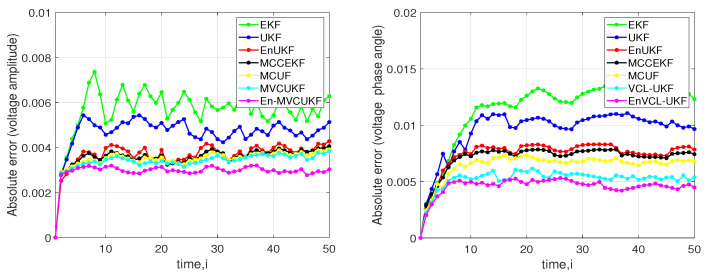
Absolute error of the voltage amplitude and voltage phase angle of the proposed methods and EKF, MCCEKF, EnUKF, UKF, and MCUF at bus 3 in an IEEE 14-bus test system under mixed-Laplacian measurement noise. (**Left**) Absolute error of the voltage amplitude; (**Right**) Absolute error of the voltage phase angle.

Secondly, due to the fact that the measurement error of real-world power flows from the voltage amplitude and voltage phase angle in power systems may follow Laplace distribution, we also incorporate the combination of Gaussian noise and Laplace noise into the IEEE-14 bus test system as the measurement noise, and we further confirm the effectiveness of the proposed methods. Specifically, the measurement noise is generated by

(57) $$\begin{array}{c} 0.85N\left(0,100\right)+0.15Lap\left(0,1\right) \end{array}$$

where $Lap\left(0,1\right)$ stands for the Laplace noise with 0 mean and scale 1. Figure 3 shows the absolute error of voltage amplitude and voltage phase angle at bus 3 in the IEEE-14 bus test system. From the simulation results, one can see that the proposed methods significantly outperform these compared KF methods in terms of the absolute error of voltage amplitude and voltage phase angle.

### 4.3. Case 2: Bad Measurement Data and Sudden Load State Change

**Figure 4:**
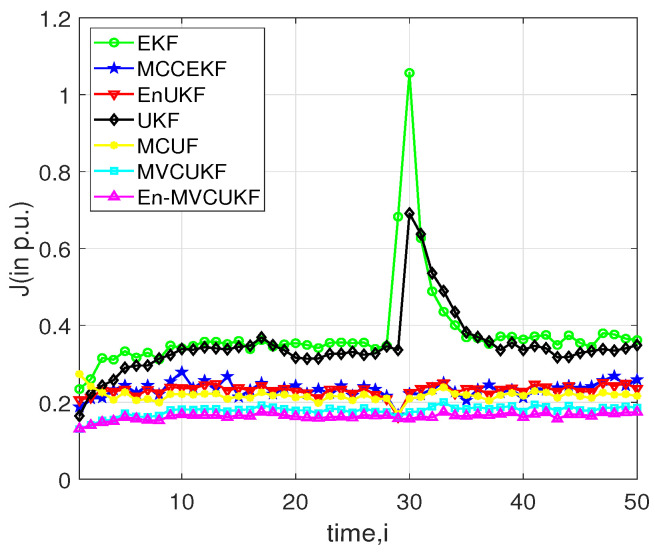
Filtering performance index *J* (in p.u.) of the proposed methods and EKF, MCCEKF, EnUKF, UKF, and MCUF in an IEEE 30-bus test system with bad measurement data at i=25.

Apart from the non-Gaussian noise with outliers in measurements, there are many undesired events that may take place in power systems for dynamic state estimation: for example, bad measurement data and sudden load state change. In order to further verify the robustness of the proposed MVCUKF and En-MVCUKF methods, this scenario is tested in which the bad data are incorporated in the measurement. Specifically, we expand 20% of the reactive power measurements at i=25 and conduct the simulation in the IEEE-30 bus test system. The overall filtering performance of all KF methods at all times is shown in Figure 4. Obviously, one can observe that from this figure, the proposed methods still have better filtering performance than other compared KF methods. Moreover, these robust KF methods, e.g., MVCUKF, En-MVCUKF, MCUF, and MCCEKF, are not sensitive to this abnormal measurement.

**Figure 5:**
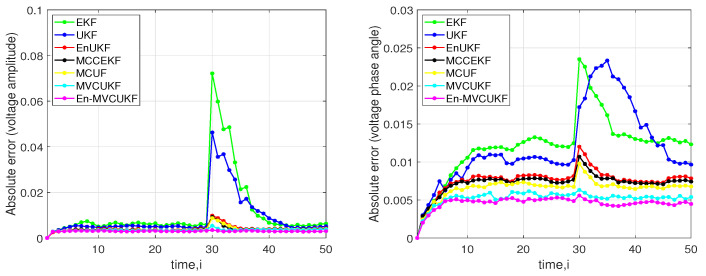
Absolute error of the voltage amplitude and voltage phase angle of the proposed methods and EKF, MCCEKF, EnUKF, UKF, and MCUF at bus 3 in an IEEE 30-bus test system with sudden load state change. (**Left**) Absolute error of the voltage amplitude; (**Right**) Absolute error of the voltage phase angle.

Then, we also tested the scenario that a sudden load change happened in the power system to study the robustness of the proposed methods. Similar to previous simulation, the IEEE-30 bus test system is utilized, and the state estimation at bus 3 is captured when the power load at bus 3 has a 20% drop at this time. The absolute error of voltage amplitude and voltage phase angle at bus 3 in the IEEE-30 bus test system are illustrated in Figure 5, in which the values of absolute error (in terms of voltage amplitude and voltage phase angle) for MVCUKF and En-MVCUKF are still lower than those of the other compared KF methods, which confirms the effectiveness and robustness of the proposed methods for dealing with the case of sudden load change.

## 5. Conclusions

In this paper, a novel unscented Kalman filter, called the maximum correntropy with variable center unscented Kalman filter (MCVUKF), is proposed for robust dynamic state estimation in power systems. Instead of using the traditional correntropy, the MCVUKF method utilizes an extended version of the correntropy that has a flexible center to unscented Kalman filter for improving the filtering performance in PSSE. In addition, an enhanced version of MCVUKF, namely En-MCVUKF, is also derived by using an exponential weight function of the innovation vector to adjust the covariance matrix for reducing the negative impact of bad data and improving the accuracy of PSSE. Extensive simulation results have illustrated that the proposed MCVUKF and En-MCVUKF methods can achieve better filtering performance for PSSE tasks in three different test systems compared with several related robust and non-robust Kalman filter approaches.

Although the proposed MCVUKF and En-MCVUKF methods have shown good results in three different test systems for PSSE tasks, they still have a limitation on selecting the optimal values for the free parameters σ and *c*. For the proposed methods, σ and *c* have great influence on the overall filtering performance for PSSE tasks. However, up to now, there has been no useful approach to select these parameters. In future work, a more effective adaptive update method will be adopted in the proposed methods to learn the optimal values for σ and *c* for further improving the filtering performance. 

## Data Availability

Not applicable.
